# Microbiota profiles in the saliva, cancerous tissues and its companion paracancerous tissues among Chinese patients with lung cancer

**DOI:** 10.1186/s12866-023-02882-1

**Published:** 2023-08-28

**Authors:** Yuhan Zhou, Hongfen Zeng, Kai Liu, Hui Pan, Baohui Wang, Minghua Zhu, Jiawei Wang, Haoyi Wang, Hongwei Chen, Dan Shen, Yue Wang, Zhaonan Yu

**Affiliations:** 1 Department of Thoracic Surgery, Sir Run Run Shaw Hospital, School of Medicine, Zhejiang University, Hangzhou City, 310000 Zhejiang Province China; 2 The First Affiliated Hospital, College of Medicine, Zhejiang University, Hangzhou, 310003 China; 3 Zhejiang hospital of Traditional Chinese Medicine, Zhejiang Chinese Medical University, Youdian Road 54, Hangzhou, China; 4 Department of Cardiothoracic Surgery, The Affiliated Hospital of Hangzhou Normal University, Hangzhou, PR China; 5 Hangzhou D.A. Medical Laboratory, Hangzhou, 310030 China

**Keywords:** Lung cancer, Saliva, Cancerous tissue, Paracancerous tissue, Microbiota

## Abstract

**Background:**

Despite the growing interest in the impact of the gut microbiome on cancer, the relationship between the lung microbiome and lung cancer has received limited investigation. Additionally, the composition of the oral microbiome was found to differ from that of individuals with lung cancer, indicating that these microorganisms may serve as potential biomarkers for the detection of lung cancer.

**Methods:**

Forty-three Chinese lung cancer patients were enrolled in the current retrospective study and 16 S rRNA sequencing was performed on saliva, cancerous tissue (CT) and paracancerous tissue (PT) samples.

**Results:**

Diversity and species richness were significantly different between the oral and lung microbiota. Lung microbiota were largely composed of the phyla Proteobacteria, Firmicutes, Bacteroidetes and Actinobacteria. The relative abundance of *Promicromonosporacea* and *Chloroflexi* increased in CT, while *Enterococcaceae* and *Enterococcus* were enriched in PT (*p*<0.05). A cancer-related microbiota model was constructed and produced an area under the curve of 0.74 in the training set, indicating discrimination between subjects with and without cancer.

**Conclusions:**

Characterization of microbiota in saliva, CT and PT from Chinese lung cancer patients revealed little difference between CT and PT, indicating that the tumor and its microenvironment might influence the local microbiome. A model to distinguish between CT and PT was constructed, which has the potential to enhance our comprehension of the involvement of microbiota in the pathogenesis of lung cancer and identify novel therapeutic targets.

## Background

Lung cancer is the leading cause of morbidity and mortality in China, with 826,000 new cases and 657,000 deaths annually [1]. Many factors, such as smoking, genetics, air pollution, occupational exposure and microbial infections, may act separately or in combination to promote its occurrence [2–4].

Past misconceptions regarding the sterility of the healthy lung have been revised as high-throughput sequencing technology has confirmed the presence of human microbes and aided in the cataloging of the human microbiome. Numerous studies have demonstrated the presence of microbiota, such as *Prevotella*, *Streptococcus*, *Veillonella*, and *Neisseria*, in the healthy lung. Changes in lung microbiota have been implicated in chronic lung diseases, such as asthma and chronic obstructive pulmonary disease [5–7]. The α- and β-diversities of the lung microbiome are believed to differ between lung cancer patients and healthy controls, with salivary *Capnocytophaga* and *Veillonella* being identified as potential biomarkers for the detection and classification of lung cancer [8–12]. The dysbiosis of lung microbiota in antibiotic-treated or germ-free mice provoke inflammation associated with lung adenocarcinoma development [13]. Anaerobic proteobacteria may colonize the tumor niche and potentiate the hypoxic tumor environment [14]. By contrast, beneficial lung bacteria may promote the effects of radiotherapy by reducing radiation-induced damage [15].

However, most conclusions regarding microbiome differences between lung cancer patients and healthy individuals have relied on the analysis of bronchoalveolar lavage fluid (BALF) samples and little is known about the influence of different sample types and locations. Therefore, the current study analyzed and compared microbiota from saliva, CT and PT in 43 Chinese lung adenocarcinoma patients who had not received any anti-infection treatment or neoadjuvant therapy prior to surgery.

## Methods

### Subject recruitment and sample collection

**Fig. 1:**
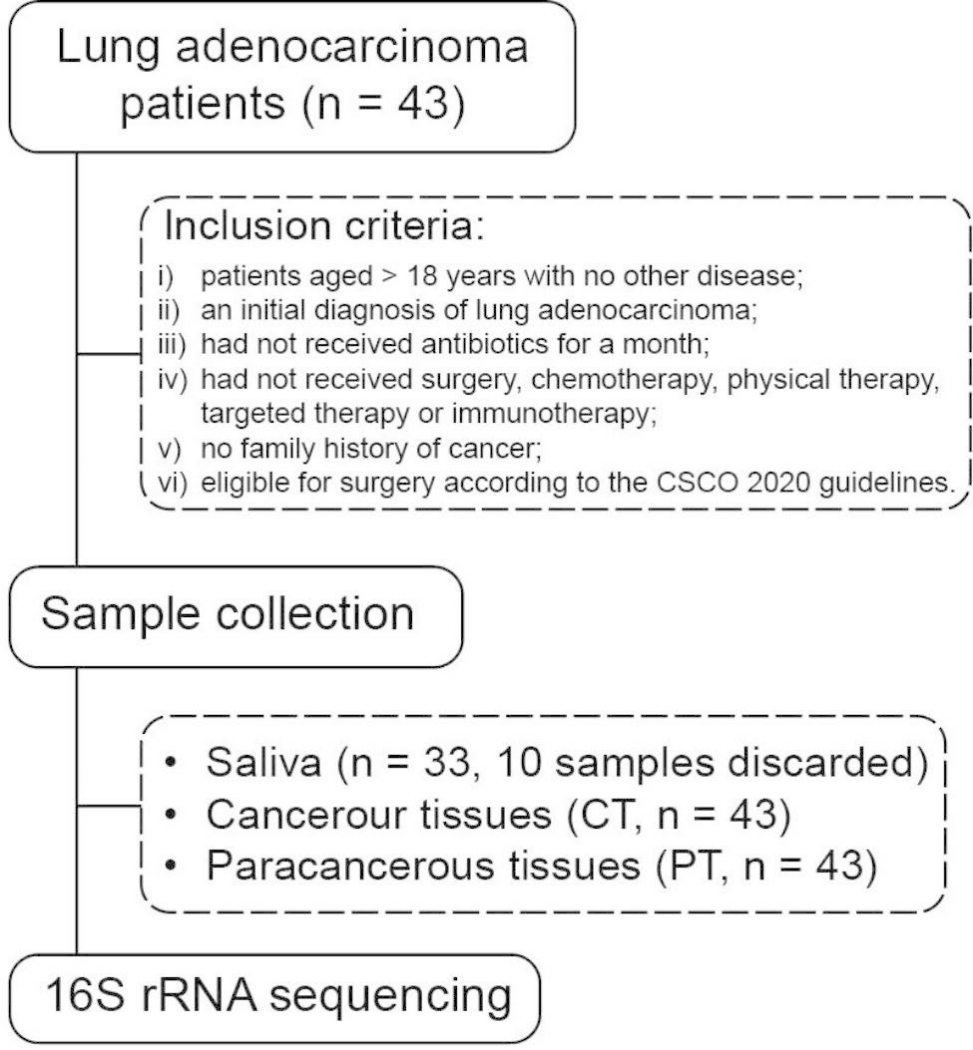
Study design and sample collection flowchart

A total of 43 lung adenocarcinoma patients presenting at Sir Run Run Shaw Hospital, Zhejiang University, China between January and December 2021 were enrolled. Inclusion criteria were as follows: (i) patients aged > 18 years with no other disease (oral disease, diabetes, hypertension.); (ii) an initial diagnosis of lung adenocarcinoma by computed tomography scan and pathological diagnosis; (iii) had not received antibiotics for a month; (iv) had not received surgery, chemotherapy, physical therapy, targeted therapy or immunotherapy.; (v) no family history of cancer; (vi) eligible for surgery according to the CSCO 2020 guidelines (Fig. 1). Demographic and clinical data, including age, gender and smoking history, were obtained from each participant.

Samples of saliva, cancerous tissues (CT) and paracancerous tissues (PT) were obtained from all patients. Saliva samples of approximately 5ml were obtained by the passive drooling method on an empty stomach before surgery. Ten saliva samples were discarded after failure of DNA extraction and quality control. CT and PT samples of approximate size, 0.5 cm × 0.5 cm, were excised during surgery and cleaned. All samples were immediately frozen in liquid nitrogen and stored at -80 °C. Ethical approval was granted by the Ethics Committee of Sir Run Run Shaw Hospital and informed consent obtained from all participants.

### DNA extraction, library preparation and sequencing

DNA was extracted using PowerMax (stool/soil) DNA isolation kit (MoBio Laboratories, Carlsbad, CA, USA), following the manufacturer’s instructions, and stored at -20°C prior to library preparation. DNA quantity was measured by NanoDrop ND-1000 spectrophotometer (Thermo Fisher Scientific, Waltham, MA, USA) and quality by agarose gel electrophoresis. Universal bacterial primers: 515F (5’- GTGCCAGCMGCCGCGGTAA − 3’) and 806R (5’- GGACTACHVGGGTWTCTAAT − 3’) were used for PCR amplification of the V4 region of 16 S rRNA to obtain an amplicon library from all samples.

### Bioinformatics and statistical analysis

Sequencing data were processed with open-source bioinformatics pipeline, Quantitative Insights Into Microbial Ecology (QIIME, v1.9.0), as previously described [16]. Sequences with low-quality (< 150 bp; Phred score < 20) were filtered and operational taxonomic units (OTUs) were picked using VSEARCH with default parameters. The representative sequence that selected from each OTU were taxonomically assigned based on the SILVA 16 S rRNA gene database (version 128). Functional profiles of differential taxa were predicted by PICRUSt (Phylogenetic investigation of communities by reconstruction of unobserved states), FAPROTAX and BugBase, as previously described [17–19].

Fisher’s exact test or Student’s t test were performed by SPSS version 24.0 (IBM Corp., Armonk, NY, USA) to analyze clinical variables. The Wilcoxon rank-sum test was performed to compare α- and β-diversity indices, bacterial abundance and function.

## Results

### Baseline characteristics of participants

Mean participant age was 63 years (range 37–77 years) and the cohort included 20 males (46.51%) and 23 females (53.49%) of whom 40 were smokers (93.02%) and 3 non-smokers (6.98%). 39 patients (90.7%) were diagnosed at stage I, 1 patient (2.33%) at stage II and 3 (6.98%) patients at stage III. No patient had received antibiotics during the preceding month. All patients had received a first diagnosis of lung adenocarcinoma and had not undergone surgery, chemotherapy, physical therapy, targeted therapy or immunotherapy (Table 1).

**Table 1:** Baseline characteristics of participants (n = 43)

Category	Status	Patient num. (prop.%)
Gender	Male	20 (46.51%)
	Female	23 (53.49%)
Smoking	Yes	40 (93.02%)
	No	3 (6.98%)
Stage	I	39 (90.70%)
	II	1 (2.33%)
	III	3 (6.98%)

### Taxonomic profiles of saliva, cancerous and paracancerous tissues

**Fig. 2:**
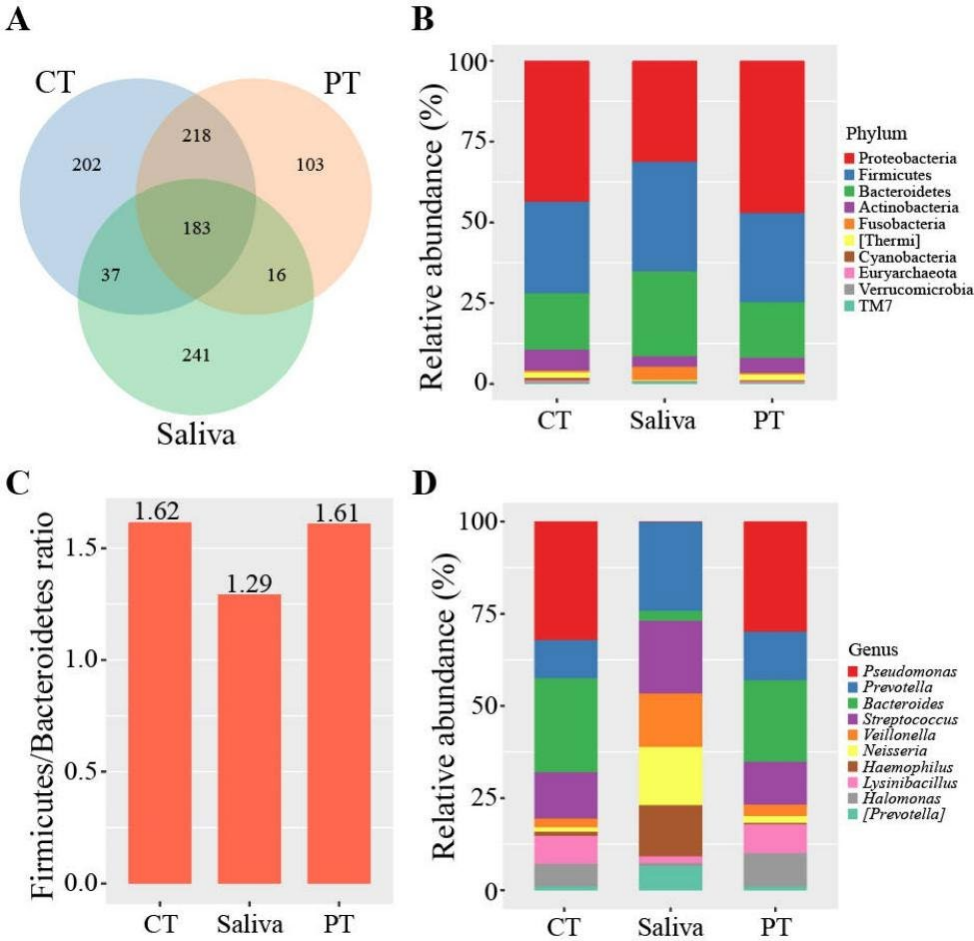
Structural composition of microbiota in CT, PT and saliva. (A) Venn diagram of OTUs in CT, PT and saliva. Blue circle means CT, red circle means PT and green circle means saliva. (B) Structural composition of microbiota in CT, PT and saliva, at phylum level. (C) Firmicutes and Bacteroidetes ratio between different sample types. (D) Structural composition of microbiota in CT, PT and saliva, at genus level

A total of 121,766 raw reads were generated from 119 samples, including 33 saliva, 43 CT and 43 paired PT samples, and processed to 111,348 high quality reads of 16 S rRNA gene (V4) region. A total of 1000 OTUs were identifiable in the samples and classified into 17 phyla, 32 classes, 51 orders, 105 families, 175 genera and 620 species. There were 183 (18.3%) core common OTUs and 241 (24.1%) OTUs exclusive to saliva, 202 (20.2%) to CT and 103 (10.3%) to PT. OTUs found to be common to 2 out of the 3 groups numbered 218 (21.8%) for CT & PT; 37 (3.7%) for CT & saliva and 16 (1.6%) for PT & saliva (Fig. 2A). Core microbiota were defined as OTUs detected in all samples in each group.

At phylum level, Proteobacteria (CT: 43.3%; PT: 47.0%; saliva: 33.6%), Firmicutes (CT: 28.1%; PT: 27.5%; saliva: 31.0%) and Bacteroidetes (CT: 17.4%; PT: 17.1%; saliva: 26.0%) were the main components, accounting for 88.8 − 91.6% of phyla detected. The fourth most abundant phylum was Actinobacteria (6.3% & 4.7%) in CT and PT but Fusobacteria (4.0%) in saliva. The Firmicutes/Bacteroidetes ratio was lower in saliva group than CT and PT, while exhibited a similar value in CT and PT (Fig. 2C). At genus level, *Pseudomonas* (CT: 12.0%; PT: 12.2%), *Bacteroides* (CT: 9.5%; PT: 9.0%), *Streptococcus* (CT: 4.7%; PT: 4.7%) and *Prevotella* (CT: 3.9%; PT: 5.4%) were predominant in CT and PT. However, the dominate genera in saliva were *Prevotella* (16%), *Streptococcus* (13.0%), *Neisseria* (10.4%) and *Veillonella* (9.6%). It may be observed that the microbiota structure of CT and PT showed a high degree of similarity but was different from that of saliva. Structural characteristics of the bacterial composition can be seen in Fig. 2B and D.

### Overall microbial richness and diversity in lung cancer patients

**Fig. 3:**
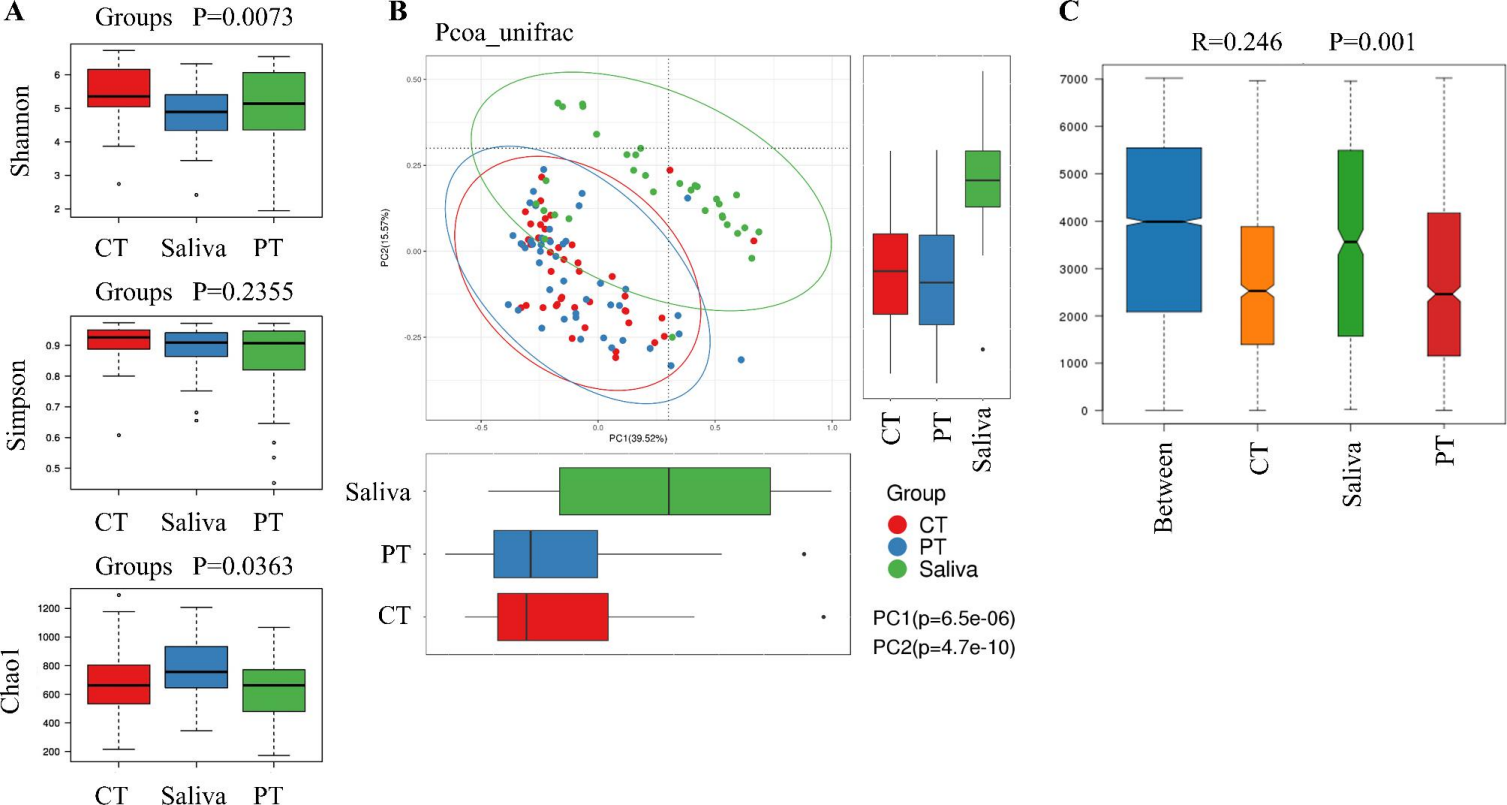
Differences of microbiota diversity among CT, PT and saliva. (A) The differences of α-diversity indices, Shannon, Simpson and Chao1, among the three groups. (B) The PCoA results with different colored dots representing the different groups. (C) Differences between and within the three groups based on ANOSIM analysis with weighted unifrac distance

The α- and β-diversities were different between the three groups. The Shannon (*p*<0.01) and Chao1 indices (*p*<0.05) were significantly different among CT, PT and saliva samples. However, no significant differences were found in the Simpson index (*p* = 0.24; Fig. 3A). The PCoA based on weighted Unifrac distance gave values for PC1 of 39.52% and for PC2 of 15.57% (*p*<0.001), although the CT and PT results were similar (Fig. 3B). Analysis of similarities (Anosim) based on weighted unifrac distance indicated significant differences among saliva, CT and PT samples (ANOSIM, R = 0.246, *p* < 0.001; Fig. 3C) with CT and PT samples showing greater similarities in microflora, due to their common origins in lung tissues, than saliva.

### Significantly enriched microbiome in cancerous and paracancerous tissues

**Fig. 4:**
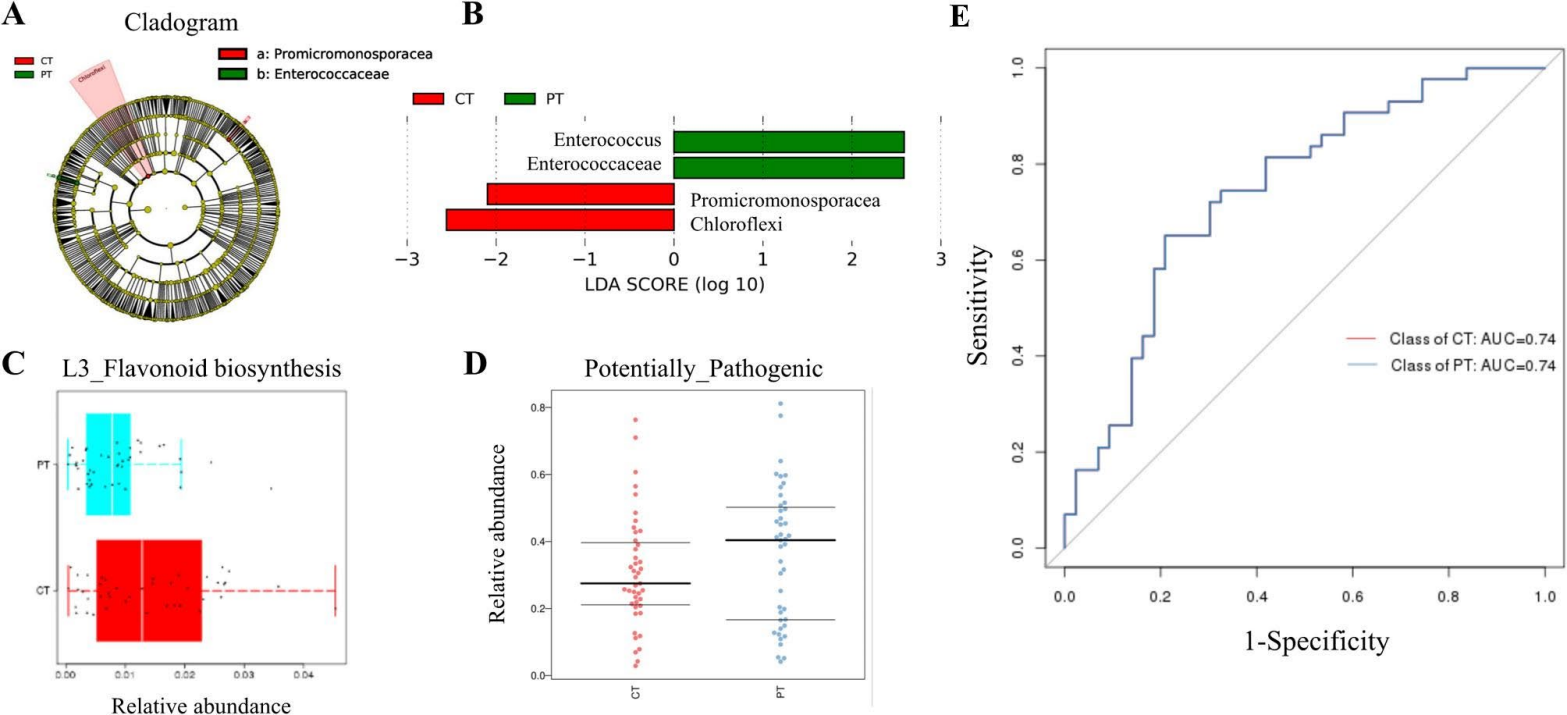
Significantly enriched microflora with relevant functions in CT and PT. Cladogram (A) and LDA histogram (B) showing microbiomes for CT and PT. Picrust (C) and Bugbase (D) predicted the functional differences in CT and PT microbiomes. ROC analysis indicated the potential diagnostic value in differentiating CT and PT (E)

LEfSe analysis was performed on the different microbiomes of CT and PT. The resulting cladogram (Fig. 4A) and LDA histogram (Fig. 4B) revealed that *Promicromonosporacea* and *Chloroflexi* were significantly enriched in CT while *Enterococcaceae* and *Enterococcus* were significantly enriched in PT. The functional significance of these differences was indicated by Picrust analysis in that flavonoid biosynthesis is likely to be higher in CT than in PT (Fig. 4C). In addition, Bugbase revealed that PT had more potentially pathogenic bacteria than CT (Fig. 4D). ROC analyses indicated that *Bdellovibrionales*, *Enterococcus* and *Desultfovibrionaceae* had the greatest impact on the specificity and sensitivity of the AUC, giving a random forest score of 69.77%. The total area under curve (AUC) of CT and PT was 0.74 (Fig. 4E).

### Co-occurrence of microbes in lung and saliva microbiomes

**Fig. 5:**
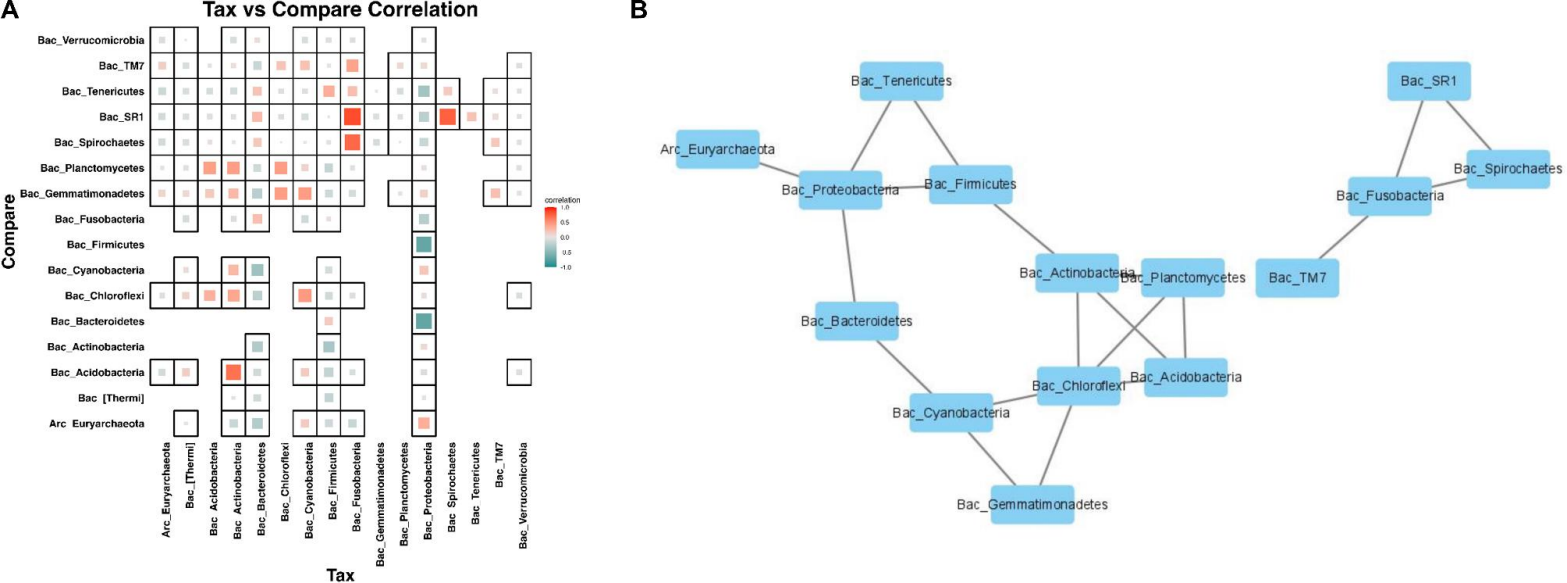
Microbial correlation network in CT, PT and saliva samples. A total of 17 phyla were analyzed

Relationships among the microbiota species were analyzed by sequencing of 17 phyla. 114 species were positively correlated and 158 were negatively correlated (Fig. 5A). Absolute values for the correlation coefficient between microbiomes of < 0.3 were disregarded, leaving 15 positive and 5 negative correlations(Fig. 5B). The strongest negative correlations between the two microbiomes were found for Proteobacteria and Bacteroidetes (-0.70) and for Proteobacteria and Firmicutes (-0.69). These 3 were the predominant phyla in samples from the 3 sources. However, Fusobacteria, the fourth dominant phylum in saliva samples, had the highest positive correlation with SR1 (0.84) and Spirochaetes (0.72).

## Discussion

Studies have demonstrated an association between the human microbiome and cancer, but the role of lung tissue and the oral microbiome in Chinese patients with lung adenocarcinoma is not fully understood. The current study first characterized the microbiota taxonomic profiles in oral saliva, cancerous and paracancerous tissues of Chinese patients with lung adenocarcinoma patients. The Shannon index of CT was significantly higher than that of PT and saliva. The increased relative abundance of *Promicromonosporacea* and *Chloroflexi*, in combination with the decreased relative abundance of *Enterococcaceae* and *Enterococcus* in lung tissues, may be associated with the risk of lung adenocarcinoma.

A NCBI literature search on the lung cancer microbiome found that the oral and lung microbiota patterns at the phylum level in this study are consistent with previous research, especially regarding Proteobacteria, Firmicutes, and Bacteroidetes [20–22]. Analysis of BALF microbiota in lung cancer samples has also shown Proteobacteria, Firmicutes, and Bacteroidetes as dominant phyla [23, 24]. However, more variation is shown in bacterial abundance at the genus level. A study of 18 non-small cell lung cancer patients found that *Pseudomonas*, *Clostridium*, and *Kocuria* were dominant genera in cancer and adjacent tissues, while *Streptococcus*, *Prevotella*, and *Neisseria* were dominant in saliva [20]. *Acinetobacter* and *Streptococcus* were the most dominant genera found in oral samples of 72 non-smoking females with lung cancer [21]. A study of 32 lung cancer patients found that *Stenotrophomonas*, *Prevotella*, *Streptococcus*, and *Haemophilus* were the most common genera in BALF samples [25]. The human microbiota is dynamic and influenced by geography, diet, host genes, and other factors, but there are core taxa and functions that remain similar across individuals [9, 26]. *Prevotella*, *Streptococcus*, and *Neisseria* are core genera in the oral microbiota of lung cancer patients, while the lung tissue microbiota shows more variation. Core genus in cancer and adjacent tissues may be influenced by tissue type (BALF or cancer tissue), primary site of lung cancer (left lung or right lung), and mutated genes. 236 common OTUs were found in oral and lung tissues, indicating an oral-lung axis. *Streptococcus*, *Rothia*, *Veillonella*, and *Prevotella* OTUs are commonly associated with poor prognosis and tumor progression in lung cancer [27]. Pathogens linked to lung cancer can spread from the mouth to the lungs and contribute to its development [28].

We found that α- and β-diversity were significantly different among saliva, CT, and PT samples. However, no significant differences in α- or β-diversity were found between CT and PT. Significant differences in α- and β-diversity have been reported in numerous tissues from lung cancer patients. However, there are no reports of differences between cancerous tissues and adjacent tissues [20, 22, 23]. Yang et al. and Lee et al. have reported differences between the two groups, but the samples used for comparison were taken from healthy individuals and patients with lung cancer [21, 24]. We believe that the differences in microbial diversity between cancer tissues and adjacent tissues of lung cancer patients were relatively small. It is possible that the microbiota of adjacent tissues was influenced by those of cancer tissues. An additional factor is that lung cancer patients often suffer from multiple lung diseases, which can lead to an imbalance in the lung microbiota.

*Fusobacterium*, a member of the phylum Fusobacteria, has been found to be enriched in saliva and has previously been linked to cancers of the lung, colon, esophagus, and pancreas [28–30]. In vitro exposure of respiratory epithelial cells to *Veillonella*, *Prevotella*, and *Streptococcus* upregulated of the ERK and PI3K signaling pathways, promoting tumorigenesis [11]. *Chloroflexi* and *Promicromonosporacea* were significantly enriched in cancerous tissues, while *Enterococcaceae* and *Enterococcus* had a higher abundance in adjacent tissues. *Chloroflexi* synthesizes extracellular polysaccharides, degrades extracellular proteins into amino acids, and plays a dual role in the initial stage of the natural nitrate cycling process [31]. *Chloroflexi* has also been reported to adhere to the tumor microenvironment in pancreatic cancer [32]. *Enterococcaceae* and *Enterococcus* are associated with enterococcal pneumonia [33]. The current patients’ medical histories revealed that almost all had previous lung complications, and it is possible that disordered lung microbiota may have preceded the development of lung cancer. Moreover, bacteria with potential pathogenic functions were enriched in adjacent tissues, while flavonoid biosynthesis was significantly higher in cancerous tissues. In general, the patients’ lungs not only contain cancer cells but also additional infections and lesions, which reveal a complex, intractable, and relapsing nature. The microbiome of paracancerous tissues may have pathogenic functions due to the spread and invasion of pathogenic bacteria from cancerous tissues. The tumor microenvironment may be composed of pathogenic bacteria and tumor cells. Flavonoid biosynthesis has been reported to have an anti-cancer effect by inhibiting the proliferation of tumor cells, exhibiting antioxidant activity, and suppressing tumor angiogenesis [34, 35]. It has been suggested that the lung microbiota contribute to lung cancer progression by effecting tumor cells or modulating the tumor-associated immune response. Jin et al. found that the lung microbiota could activating lung-resient γδ T cells to promote inflammation and tumor cell proliferation [13].

The present microbiome analysis was conducted to identify biomarkers for lung cancer. Ten genera, including *Pseudomonadaceae*, *Capnocytophaga*, *Stenotrophomonas*, *Microbacterium*, *Gemmiger*, *c:TM7-3*, *Oscillospira*, *Blautia*, *Lautropia*, and *Sediminibacterium*, showed differential abundance and produced an AUC of 79.12% (95% CI: 66.41–91.83%) in distinguishing between lung cancer patients and those with benign pulmonary diseases [12]. Two previous studies that examined the diagnostic potential of lung cancer biomarkers reported AUCs of 0.693 and 0.888 [8, 24]. We believe that our study is the first to conduct ROC analysis of biomarkers that enable the differentiation between lung cancer tissues and adjacent tissues within lung cancer patients. The sensitivity and specificity of the diagnostic model require urgent improvement through the inclusion of more microbial data from patients with lung cancer. Recent studies have demonstrated that the lung microbiota can potentially function as biomarkers for the prediction of lung cancer stages, particularly when combined with transcriptome information [36]. However, the present study did not yield a similar outcome, possibly due to the limited sample size utilized in the research.

Network analysis illustrates competition and cooperation among microbes. The ecological theory developed by Coyte et al. describes the benefits to hosts of microbial stability resulting from the promotion of microbial competition and the weakening of ecological interactions [37]. In the present study, it was found that Proteobacteria have a negative correlation and are considered to play a role in host immunity by competing with pathogenic bacteria [38, 39]. In addition, the positive correlations found between Fusobacteria and colorectal cancer, and between SR1 and liver cancer, respectively, are consistent with their known roles [40, 41].

The concept of “One Health Care for the Oral-Lung Axis” pertains to the interplay between the host (genetic factors, aging and immunity, population susceptibility), environmental changes, and microbiome (balance and disorder). This approach aims to preserve the overall health of living organisms. The microbiome is also a potential target for treating lung cancer, and fecal microbiota transplantation or probiotic therapies are potential strategies for achieving this goal. Fecal microbiota transplantation has been successfully used in two groundbreaking clinical case studies involving prostate cancer and metastatic urothelial carcinoma [42]. Probiotic therapy, including the Clostridium butyricum MIYAIRI 588 strain, had a positive impact on the therapeutic efficacy of immune checkpoint blockade in patients with non-small cell lung cancer [43]. The act of smoking has been identified as a significant contributor to the development of lung cancer. Furthermore, research has revealed a correlation between an individual’s smoking history and the composition of their lung microbiota. According to the study conducted by Vogtmann et al., individuals who smoke exhibited a higher relative abundance of Lactobacillus in comparison to those who have never smoked [44]. We look forward to further research on microbial therapy for cancer treatment.

## Conclusions

In summary, the microbiota of lung cancer patients exhibited notable differences between CT and PT. The microbial communities in CT and PT were found to differ significantly, with *Promicromonosporacea* and *Chloroflexi* being predominant in CT, while *Enterococcaceae* and *Enterococcus* were significantly enriched in PT. A random forest model with microbiota information has successfully distinguished CT and PT samples, leaving *Bdellovibrionales*, *Enterococcus*, and *Desultfovibrionaceae* as bacterial biomarkers. The present study elucidates the role of microbiota in the incidence and progression of lung cancer. In this study, however, microbiome annotations were limited to the class and family levels, and the individual microorganisms were not annotated beyond the genus or species level. High-throughput sequencing based on the V4 region of the 16 S rRNA gene enables only limited classification of bacterial categories and functions. Metagenome sequencing or sequencing the entire length of the 16 S rRNA gene may offer more accurate insights in the future. These advanced technologies make it possible to access further genetic information of the microbiome, paving the way for more meaningful discoveries. Meanwhile, the current study had the drawback of a limited sample size, and we plan to collect additional samples in the future to address this limitation.

## Data Availability

The 16 S sequencing data were deposited in China National GeneBank DataBase under Accession CNP0003512 (https://db.cngb.org).

## List of Abbreviations

CT: Cancerous tissue
PT: Paracancerous tissue
BALF: Bronchoalveolar lavage fluid
QIIME: Quantitative insights into microbial ecology
OTUs: Operational taxonomic units
PICRUSt: Phylogenetic investigation of communities by reconstruction of unobserved states
PCoA: Principal coordinates analysis
Anosim: Analysis of similarities
LEfSe: Linear discriminant analysis Effect Size
ROC: Receiver operating characteristic
AUC: Area under curve
